# CD33 splice site genotype was not associated with outcomes of patients receiving the anti-CD33 drug conjugate SGN-CD33A

**DOI:** 10.1186/s13045-019-0771-0

**Published:** 2019-08-22

**Authors:** Michele Stanchina, Alessandro Pastore, Sean Devlin, Christopher Famulare, Eytan Stein, Justin Taylor

**Affiliations:** 1 Human Oncology and Pathogenesis Program, Memorial Sloan Kettering Cancer Center, New York, NY USA; 2 Department of Biostatistics, Memorial Sloan Kettering Cancer Center, New York, NY USA; 3 Center for Hematologic Malignancies, Memorial Sloan Kettering Cancer Center, New York, NY USA; 4 Leukemia Service, Department of Medicine, Memorial Sloan Kettering Cancer Center, 1275 York Avenue, New York, NY 10065 USA

**Keywords:** Acute myeloid leukemia, CD33, Antibody-drug conjugate

## Abstract

We tested whether a single nucleotide polymorphism (SNP) that affects splicing of CD33 predicted response to treatment in adults with acute myeloid leukemia (AML) who received the novel CD33 antibody-drug conjugate SGN-CD33A. This genotype, for the CD33 splice site SNP rs12459419, was not associated with clinical response (30% CR/CRi in both groups), event-free survival, or overall survival.

## Main

Antibody-drug conjugates (ADCs) are among the most promising immunotherapies developed in the last few decades for patients with acute myeloid leukemia (AML) [1]. The CD33 antigen (SIGLEC-3) is highly expressed on AML blasts and has been a popular target for immunoconjugate drugs, as well as unconjugated antibodies and radioimmunotherapeutics [2]. Gemtuzumab ozogamicin (GO), a humanized anti-CD33 monoclonal antibody conjugated to the cytotoxic agent calicheamicin, first demonstrated the potential efficacy of targeting CD33; it was most effective in patients with favorable-risk cytogenetics [3, 4] and higher expression of CD33 [5–8]. In 2017, Lamba and colleagues found an association in pediatric AML patients between response to GO plus chemotherapy and genotype at a single nucleotide polymorphism (SNP) in *CD33* [9]. This SNP (rs12459419) occurs at a splice site of the *CD33* gene and affects the expression of the extracellular epitope recognized by GO. Variation from the common C allele to the rare T allele abrogates the splice site for inclusion of exon 2, which codes for the IgV domain of CD33; without the C allele, the exon is skipped during transcription. Thus, there is a plausible biological mechanism for altered response to GO. However, when Gale and colleagues performed a similar analysis in adult patients with AML treated with GO plus chemotherapy, they did not find an association between the SNP and outcomes [9, 10]. Furthermore, there is no data for CD33-targeted agents beyond GO. We aimed to assess the association between this SNP and the efficacy of CD33-targeting in a cohort of adults (age ≥ 18 years) with AML. Our patients were treated with an alternative ADC directed against CD33, SGN-CD33A, a monoclonal anti-CD33 antibody conjugated to a pyrrolobenzodiazepine (PBD) dimer.

**Fig. 1:**
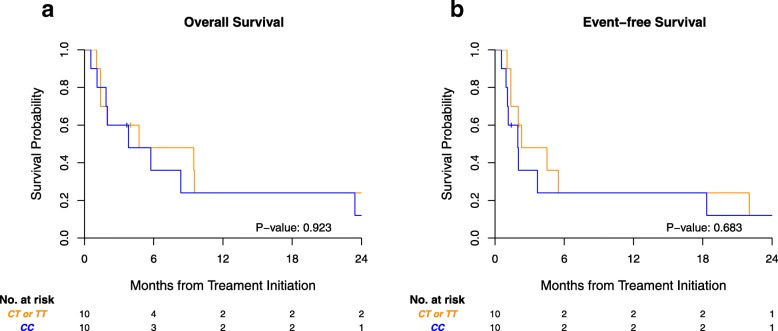
No difference in outcome according to CD33 splice site genotype for patients receiving SGN-CD33A. Kaplan-Meier survival estimates for **a** overall survival and **b** event-free survival

Twenty patients with CD33+ AML who received SGN-CD33A either as monotherapy (10–50 mcg/kg) or in combination with hypomethylating agents (10 mcg/kg SGN-CD33A and standard doses of hypomethylating agent) were tested for the CD33 SNP genotype (rs12459419) using TaqMan SNP genotyping (Applied Biosystems, CA). Clinical characteristics of disease, prior treatments, and outcome data were collected and analyzed for association of the SNP genotype with response rate, the primary objective. Event-free and overall survivals were secondary objectives assessed by the Kaplan-Meier estimator. We included adults with de novo and secondary AML who had either experienced disease relapse or declined intensive chemotherapy. Much as would be expected at the population level, we saw a 50%/40%/10% distribution of genotypes CC, CT, and TT, respectively. The CT and TT genotypes were combined in our analysis because of the low numbers of TT genotype (*n* = 2) and the previously reported decreased response to anti-CD33 ADCs in patients carrying even one T risk allele [9]. Baseline characteristics by genotype are shown in Table 1. There was no significant difference in response between patients carrying the most common genotype, CC (*n* = 10), and those carrying CT or TT genotypes (*n* = 10): both groups had 30% complete response (CR) or complete response with incomplete hematologic recovery (CRi). The genotype for the CD33 splice site SNP rs12459419 was also not associated with event-free survival or overall survival (Fig. 1).

**Table 1:** Characteristics of patients treated with SGN-CD33A by genotype

	CC (n=10)	CT/TT (n=10)	Total (n=20)	*p* value
Gender				0.361
Female	5 (50.0%)	7 (70.0%)	12 (60.0%)	
Male	5 (50.0%)	3 (30.0%)	8 (40.0%)	
Age				0.418
Mean (range)	67.3 (27.5-80.0)	72.3 (42.0 -82.6)	69.8 (27.5-82.6)	
ECOG				0.160
0	2 (20.0%)	5 (50.0%)	7 (35.0%)	
1	8 (80.0%)	5 (50.0%)	13 (65.0%)	
Risk group				0.148
Adverse	7 (70.0%)	4 (40.0%)	11 (55.0%)	
Favorable	1 (10.0%)	0 (0.0%)	1 (5.0%)	
Intermed.	2 (20.0%)	6 (60.0%)	8 (40.0%)	
BM blast **%**				0.334
Median (Q1, Q3)	64.0 (43.2, 80.0)	54.0 (24.0,66.0)	56.0 (36.0,78.0)	
WBC				0.200
Mean (SD)	13.6 (17.1)	5.6 (8.1)	9.6 (13.7)	
Median (Q1, Q3)	5.3 (1.7, 16.5)	1.7 (1.2, 3.8)	2.5 (1.5, 16.4)	
Range	0.8 - 51.7	0.4 - 23.1	0.4 - 51.7	
IQR	14.8	2.7	14.9	
Platelets				0.913
Mean (SD)	50.3 (41.2)	52.4 (43.9)	51.4 (41.5)	
Median (Q1, Q3)	34.0 (22.0, 75.0)	32.0 (21.8,90.8)	34.0 (19.8,89.5)	
Range	11.0 - 133.0	5.0 - 116.0	5.0 - 133.0	
IQR	53.0	69.0	69.8	
De novo				0.531
Yes	1 (10.0%)	2 (20.0%)	3 (15.0%)	
No	9 (90.0%)	8 (80.0%)	17 (85.0%)	
Line of tx				0.361
1st	3 (30.0%)	5 (50.0%)	8 (40.0%)	
2nd	7 (70.0%)	5 (50.0%)	12 (60.0%)	

*BM* bone marrow, *ECOG* Eastern Cooperative Oncology Group, *IQR* interquartile range, *tx* treatment, *WBC* white blood cell

While limited by the small sample size of this study, our data show that genotype of the CD33 splice site SNP was not associated with outcomes of patients treated with an anti-CD33 drug conjugate, aligning with previously reported data for adult patients [10] and extending this finding to the novel ADC SGN-CD33A. The fragment variable (Fv) regions of SGN-CD33A and GO recognize the same epitope on CD33, so any effect of the CD33 splice site SNP is expected to be similar for both agents. While the drug payload conjugated to CD33 differs between GO and SGN-CD33A, both are very potent agents unlikely to produce significantly different efficacy. However, published studies of GO have included different patient populations and treatment combinations that may account for disparate results between pediatric and adult populations. Compared with patients in previous studies, our patients were generally older and were not treated in combination with chemotherapy. Notably, *CD33* SNPs are germline mutations, so these could result in different expression of CD33 in off-target tissue. An alternative hypothesis, therefore, is that increased toxicity in the CC genotype would offset the drugs’ benefit; however, neither our study nor the other adult study showed any difference in response rates between genotypes. This lack of any benefit for the CC genotype in adult AML suggests that age-related or other biological differences between adult and pediatric AML may explain disparate results between these groups.

This study suggests that *CD33* genotype is a poor biomarker for broad use in adults with AML to predict response to CD33-targeted ADCs. While these results are disappointing—because genotype is much more reliably measured, reported, and interpreted than CD33 expression by flow cytometry, another potential biomarker for anti-CD33 ADC response—larger studies may nonetheless show a benefit for genotype testing in specific patient subsets identified by age, disease risk, or mutational subtype.

## Data Availability

The datasets used and/or analyzed in the current study are available from the corresponding author on reasonable request.

## Abbreviations

ADC: Antibody-drug conjugate
AML: Acute myeloid leukemia
CR: Complete response
CRi: Complete response with incomplete hematologic recovery
GO: Gemtuzumab ozogamicin
SNP: Single nucleotide polymorphism
